# Hospital Funds and the Working Classes

**Published:** 1887-01-08

**Authors:** Alderman Fred. R. Spark

**Affiliations:** Leeds


### Hospital Funds and the Working Classes.

By Alderman Fred. R. Spark, Leeds.

Health, the Workman's Capital.

A new movement, which promises to be a great success, has been started in Leeds for the purpose of increasing workpeople's contributions to the medical charities, by means of a thorough organisation permeating every part of the wide borough, and embracing every work-place, from the largest mill and factory to the smallest home workshop. At the public meetings held in furtherance of this object, much valuable information, numerous facts and figures, and many novel ideas have been imparted. To the working-classes it has been pointed out how the medical charities serve them in a direct financial sense; and the object of this paper is to emphasise the fact that to hand-workers a physical capacity for daily labour is their chief, if not their sole, capital. Without health, without strength, minus a limb, sight, or hearing, a labourer is deprived of his usual means of livelihood. Any plan, or any treatment, therefore, which restores to him his ability to labour, is tantamount to investing him with capital; and any scheme by which he can recover from disease or accident, and which enables him to reduce the ordinary time of his enforced absence from work, is just so much pecuniary gain to him. This aspect of medical institutions has not been sufficiently regarded by the working-classes, whose contributions to the funds of our hospitals are by no means as large as probably they would be, were this view of the question forcibly placed before them.

To a large extent the amount of money saved by workpeople in this direction can only be matter of conjecture; yet, from a certain standpoint we may calculate pretty fairly what a man may save—or, rather, what he may gain—by medical treatment in a well-appointed hospital, in place of the ordinary treatment in his own home.

Financial Advantage of Quick Recovery.

Take, for instance, the case of a workman who meets with a serious accident: say the crushing of a leg or an arm—a common occurrence in a large manufacturing town. Follow this man to his home—a small house, with one sitting-room and (say) three bedrooms. His wife has more than enough to do to keep the house in order, and to look after the children. The husband, sorely injured and perfectly helpless, is placed in the "best" bedroom. Under the most favourable circumstances, the attention and attendance he receives will be poor and inadequate; the appliances for his recovery will be meagre. In addition, the breathing-space for the man is contracted, and the atmosphere is polluted. Bad sanitation, and consequently bad air, are terrible drawbacks to a patient's recovery. These facts considered, it may not be estimating too high the time required for his recovery at double that which would probably be sufficient were the sufferer a patient in a public hospital. Here he has every attention, every known appliance, which may conduce to his speedy recovery. A spacious ward, pure air, constant nursing by night and by day, proper food prepared under medical direction, and surgical ability of the highest character. In fact, the workman patient receives such care, such united skill, and such scientific treatment, that it may be doubted whether the wealthiest person could obtain its counterpart in his own house.

Great Saving of Time.

Moderately computed, I estimate that a workman's recovery will take place in one-half the ordinary time when he is treated in a hospital. And even in these institutions there is considerable variation in the period of residence, arising from the condition of the building itself, the situation, and the mode of treatment. The present most excellent Leeds Infirmary retains its in-patients for an average period of three weeks only. In earlier days, when the Infirmary was situated in a different part of the town, and when medical science had not attained its present high position, the mean residence of each in-patient ranged from thirty-two to thirty-five days. If, then, the period of incapacity to work be reduced from six weeks to three, what an enormous gain must this be to the working-classes! During the year 1885, no fewer than 4,400 patients were treated in the Leeds Infirmary. Of these, we may calculate that 3,000 were adult workpeople, each of whom is enabled, by superior treatment, to work three weeks more than if no such institution existed. Here, then, we have by means of this one hospital an aggregate of 9,000 weeks restored to hand-labour. Calculated at only 10*s.* per week each person, this represents a saving, or a gain, of £4,500 in Leeds alone. Nor is this all, for a sum equally as large is also saved by the local Friendly Societies and Trade Unions, out of whose funds the disabled workman is maintained for a period of three weeks only, instead of six, as a consequence of these medical institutions.

Do Contributions give Rights?

By placing the great advantages of the modern hospital prominently before the working-classes, there may be, in the opinion of many who take deep interest in these institutions, danger lest those who can afford personally to pay for medical attendance should prefer to take advantage not only of their superiority, but of their charitable side, claiming this as a right, because they subscribe their weekly pence. And this aspect of the case has been set forth in the Leeds press during the progress of the new movement to which I have alluded. It is to be regretted that this view should, at such a time and in such a connection, have been promulgated; for if the working-classes are to be credited with an unworthy motive because they may subscribe liberally to the medical charities, then it were better if they were not asked to subscribe anything at all. But it is a mistake to suppose that the working-classes do not make provision for ill-health, and that they subscribe—at least, the best of them—to the medical charities from mere mercenary motives. It is estimated that there are no fewer than four million members of Friendly Societies in the United Kingdom. The average amount paid annually out of the Sick Fund of these Societies to each member may be taken at 14*s.* This gives a total sum of no less than £2,800,000. Of this total, about two per cent. may be credited to accidents; so that the annual sum of £56,000 is paid by these Societies for this class of bodily ailment. Now if, by the action of hospitals throughout the United Kingdom, members of Friendly Societies who meet with accidents recover in one-half the ordinary time, it follows that no less a sum than £28,000 per year is saved by these Societies as a consequence.

Sums Paid by Workpeople.

It has been urged in some quarters that medical men are considerable losers by the existence of medical charities, whose doors are open to the working-classes in receipt of weekly wages. Some idea, however, may be formed of the large sums paid to the medical profession on behalf of weekly wage-earners, from the fact that the Ancient Order of Foresters alone paid for medical aid to its members in the United Kingdom during the year 1885, no less a sum than £74,818. The Manchester Unity of Oddfellows paid in the same year about a similar amount; and it may be safely asserted that the whole of the Friendly Societies pay no less than £550,000 per year for medical attendance and for medicine. In Leeds the Friendly Societies pay about £5,000 per annum to their medical attendants. With these facts in view, it cannot, I think, be contended that working people are indifferent to "the ills which flesh is heir to," or that they do not adequately provide for the proverbial "rainy day." The fears, therefore, of those who imagine that gifts from workpeople to the medical charities may constitute a right to claim help from those charities, are utterly groundless. So far as Leeds is concerned, there has not been urged at any of the numerous meetings of workpeople held in the various wards a single idea which could be twisted into a right to share the benefits of the medical charities; although many a speaker has urged that when accident or complicated disorder should compel a workman to seek the especial surgical ability of a hospital, he would become a patient with less compunction, if he has been a regular subscriber to its funds, than if he had neglected his duty whilst in a position to subscribe.

A Hopeful Conclusion.

It may then be safely asserted, that whilst to the working-classes hospitals are of immense benefit, both from a physical and a financial point of view, yet these classes are by no means destitute of a practical sympathy with those institutions, which sympathy will induce many of them to join such a grand movement as that we have just established in Leeds.

(*To be continued.*)

### MEDICAL.

It is noticeable that when a patient begins to feed more, his doctor is always *feed* less.

